# Reflexive Saccades Used for Objective and Automated Measurements of Contrast Sensitivity in Selected Areas of Visual Field

**DOI:** 10.1167/tvst.11.5.29

**Published:** 2022-05-31

**Authors:** Peter Essig, Yannick Sauer, Siegfried Wahl

**Affiliations:** 1 Institute for Ophthalmic Research, University of Tübingen, Tübingen, Germany; 2 Carl Zeiss Vision International GmbH, Aalen, Germany

**Keywords:** contrast sensitivity, visual field, saccades, eye tracking, psychophysics

## Abstract

**Purpose:**

This study proposes a novel approach for objective and automated peripheral contrast sensitivity (CS) testing using reflexive saccades. Here the CS was examined in various areas the of visual field (VF) using a live analysis of gaze data. For validation of the new test, we examined CS with an established procedure of identifying the orientation of a contrast stimulus.

**Methods:**

To perform and validate the saccade-based testing, two separate measurement events were performed. In the first, participants were asked to execute a saccade toward a newly-appeared stimulus in their VF. After the saccade execution or stimulus expiry, reporting the target orientation was required in a four-alternatives forced choice (4AFC). Therefore the first measurement yields two outcomes (objective and subjective). In the second measurement, only the identification of the stimulus orientation was requested, while fixating a central mark. Stimulus contrast was controlled by an adaptive psychometric procedure in both measurements.

**Results:**

The study found strong correlations (all *r* ≥ 0*.*79) of CS values for all three possible testing methods (saccade-based responding in saccadic measurements, keyboard-based responding in saccadic measurements, keyboard-based responding in non-saccadic measurements), showing the feasibility of employment of reflexive saccades in such testing. Second, this study shows a significant influence of eccentricity and direction of the stimulus on the CS function.

**Conclusions:**

CS measured with reflexive saccades is comparable to other testing methods over several areas of the participant's VF. Hence, we propose it as a novel and objective testing procedure for CS measurements.

**Translational Relevance:**

Assessment of CS using reflexive saccades extends the portfolio of suggested eye movement-based tests, allowing objective examination across the VF, which might be helpful especially in the early detection of various eye diseases.

## Introduction

Objective assessment of visual performance is possible using several types of eye movements. Previous research showed a successful implementation of microsaccades,^1^ smooth pursuit eye movements,^2^ or the optokinetic nystagmus^3^^–^^5^ in contrast sensitivity (CS) measurements. It has been already stated that eye movement-based measurements may help to examine non-communicative participants,^2^ and make the measurements more time-efficient when using a live gaze data analysis.^4^ Their utilization is considered an objective approach for visual performance testing.^1^^–^^4^^,^^6^^,^^7^ However, by now, these methods measure only the central CS, whereas an eye movement-based test for peripheral CS has not been developed yet.

Peripheral vision is extensively used in patients with central visual field loss resulting from macular diseases.^8^ Moreover, a reduction in peripheral vision may be the first indication of serious eye diseases, such as glaucoma. Next, the optical quality on the peripheral retina might contribute to the enigmatic myopia development process.^9^ Last, good peripheral vision is important for many daily tasks, such as scene recognition,^10^ driving^11^ or performing sports activities.^12^ In light of these facts, we aimed to transfer the advantages of an eye movement-based visual performance test to the examination of peripheral CS. To reach this goal, we use live detection (live gaze data analysis) of reflexive saccades (visually-guided eye movements), which are performed in the direction of a newly appeared visual stimulus, as an objective eye movement-based response.^13^ Searching for the contrast level at which a reflexive saccade just occurs could be therefore used as an objective measure of peripheral CS. To effectively change the contrast level of the visual stimulus while searching for the contrast threshold, an adaptive psychometric procedure was applied.

As shown by Murray et al.,^14^ or more recently by Perperidis et al.,^15^ reflexive saccades were successfully used for perimetry testing (SVOP technique). The motivation of the current study was to extend the portfolio of eye movement-based visual field tests to examinations of peripheral CS. The already established eye movement-based procedures, along with our newly proposed one, could serve as a set of tests for objective assessment of visual performance across the visual field of a patient.

Reflexive saccades are controlled by different neural structures than voluntary saccades.^16^ Moreover, the latency of the reflexive saccades was found to be lower (the eye movement was performed faster) compared to voluntary saccades.^13^ Similar to optokinetic nystagmus,^17^ the onset time of reflexive saccades is influenced by the contrast level of a stimulus.^18^ Therefore we used the visual stimulus presentation time limit of 500 ms, which was shown to cover all the onsets across a wide range of contrast levels.^18^ Reflexive saccades are often also referred to as reactive saccades. Throughout the article we use the term *reflexive saccade* to keep this work uniform.^13^^,^^19^^,^^20^

Because previous subjective examinations of peripheral CS showed varying values of CS depending on the eccentricity and direction (nasal, temporal, inferior, and superior),^21^ we aimed to replicate this VF location-specific contrast sensitivity function (CSF) for the objective measurements. Location dependent variations of CS have been explained by variations in peripheral refraction, higher-order aberrations, and asymmetrically decreasing sampling of the ganglion cells toward the periphery.^22^ To validate our proposed objective test, we also executed the subjective measurements. In the subjective measurements, participants were asked to respond to the perceived orientation of a Gabor patch with a keyboard. This subjective procedure was performed twice: once in the saccade-based measurements directly after performing a saccade (or after stimulus expiration), and once in a separate measurement, while maintaining the fixation in the center of the screen (non-saccadic measurements). To keep uniform references to the measurement instances we performed, we address them as saccade-based responding in saccadic measurements, keyboard-based responding in saccadic measurements and keyboard-based responding in non-saccadic measurements.

CS is clinically tested under monocular viewing conditions. Hence, we followed this approach by patching the left eye. Moreover, we used one fixed stimulus size as the potential effect of cortical magnification varies substantially among subjects and visual field^23^ and one stimulus size for all tested eccentricity levels was used in a previous study.^21^ Furthermore, as the current study aimed to test CS in different visual field areas, namely the central, macular and near peripheral, the target was presented accordingly displaced relative to the center of the screen. The estimated ranges of the respective visual field areas are <5°, ≈5° to ≈9° and ≈9° to ≈17°, as reviewed by Strasburger et al.^24^

In our study, the contrast thresholds were measured independently over a selected range of spatial frequencies at four cardinal directions in three eccentricity levels. These measurements were conducted using saccade-based and keyboard-based procedures.

## Methods

### Participants

Twelve participants (eight male and four female) with a mean age of 24.5 ± 2*.*6 took part in the current study. All participants were emmetropic and had normal vision. The current study considered emmetropia as a refractive error smaller than ±0*.*5 D in spherical equivalent measured by the wavefront-based autorefraction (ZEISS i.Profiler plus, Carl Zeiss Vision, Aalen, Germany). The study protocol followed the Declaration of Helsinki. In addition, the study was approved by the ethics committee of the Faculty of Medicine of the University Tuebingen. Signed informed consent was obtained from all participants before the experiment. All participants were recruited from the University Tuebingen.

### Visual Stimulus and Eye Tracking

For the presentation of the visual stimulus, the Viewpixx screen (VIEWPixx; VPixx Technologies Inc., Saint Bruno, Quebec, Canada) was used, providing 12 bits of bit depth in gray-scale resolution and refresh rate of 120 Hz. The screen was gamma-corrected to compensate for the nonlinearity in the pixel value and luminance. For eye tracking, the EyeLink 1000 Plus infra-red (IR) eye-tracker (SR Research, Ottawa, Ontario, Canada) was used with monocular tracking of the right eye with a sampling frequency of 1000 Hz. For the visual stimulus creation and data analysis MATLAB2018b (MATLAB2018b; MathWorks, Natick, MA, USA) and Psychtoolbox-3^25^^,^^26^ were used. The reflexive saccade-evoking stimulus was a Gabor patch with a diameter of 1*.*4°, presented on a screen at a distance of 62 cm to the participant's right eye (left eye was patched for monocular testing). The stimulus size was chosen to be large enough to not influence the detection performance across a wide range of eccentricities.^27^ Also, the size was fixed for all eccentricity levels because the cortical magnification, and thus the related retinal cone density,^28^ was expected to vary among subjects and to be different in the four tested directions. One constant stimulus size for different eccentricities was used in a previous study on peripheral CS as well.^21^ During one measurement, the target was presented in a random order in one of the four possible directions respecting the nasal, temporal, inferior, and superior visual field; hence, always displayed at the 0°, 90°, 180°, or 270° meridian. The orientation of the target was also randomly selected for every target presentation from four available orientations 45°, 90°, 135°, or 180°. Each measurement was performed separately for three predefined eccentricity levels, matching the central, macular, and near-peripheral visual field. Here the corresponding displacements of the target relatively to the center of the screen were 2*.*0°, 6*.*5°, and 11*.*0°. The selection of tested spatial frequencies was dependent on the eccentricity level. We aimed to test the relevant range around the peak of the CSF for each eccentricity level, also considering the hardware limitations of our setup. We tested spatial frequencies 0.8, 1.4, 2.2, and 4*.*3 cycles per degree (cpd) for all three eccentricity levels, with one additional (7*.*2 cpd) for the macular eccentricity level and two additional (7.2 and 10*.*7 cpd) for the central eccentricity level, because the detection of high spatial frequencies is too low in the near peripheral eccentricity level.^21^ To ensure stimulation at the desired visual field location, the participants’ gaze was controlled to be on the central fixation mark before the visual stimulus was presented. This fixation check was implemented using live analysis of gaze data and continuous calculations of the gaze position running with the sampling rate of the eye-tracker. A gray dot, displayed at the center of the screen in size of 0*.*3°, served as the fixation mark. The radius of the fixation area was set to 1° so that potential fixational eye movements would not be accidentally detected as false-positive responses in the saccade-based measurements.^7^^,^^29^ Furthermore, the fixation phase varied in duration because the time was randomly selected from a range between 500 to 650 ms in every trial. Live gaze analysis allowed us to switch to an irrelevant stimulus (empty circle) as soon as the gaze position had exceeded the fixation area and, second, to let a particular trial be repeated if a blink was detected or if inappropriate fixation was detected in the keyboard-based measurements. In case of a blink, the order of the remaining trials was randomized again. In the saccade-based measurements, as soon as the gaze was found to be out of the fixation area, the direction of the performed saccade (*β_g_*) was calculated as follows:

(1) 12βg=atan2yeye-ycenter,xeye-xcenter

In Equation 1 *x*_eye_ and *y*_eye_ stand for the Cartesian coordinates of the gaze position in pixels captured after exceeding the fixation area, and the variables *x*_center_ and *y*_center_ are the coordinates of the center of the screen. The calculated angular difference between target location and saccade direction β*_g_* had to be in a range of ±22*.*5° to be accepted as a correct response.

### Task for the Participants

**Figure 1.**
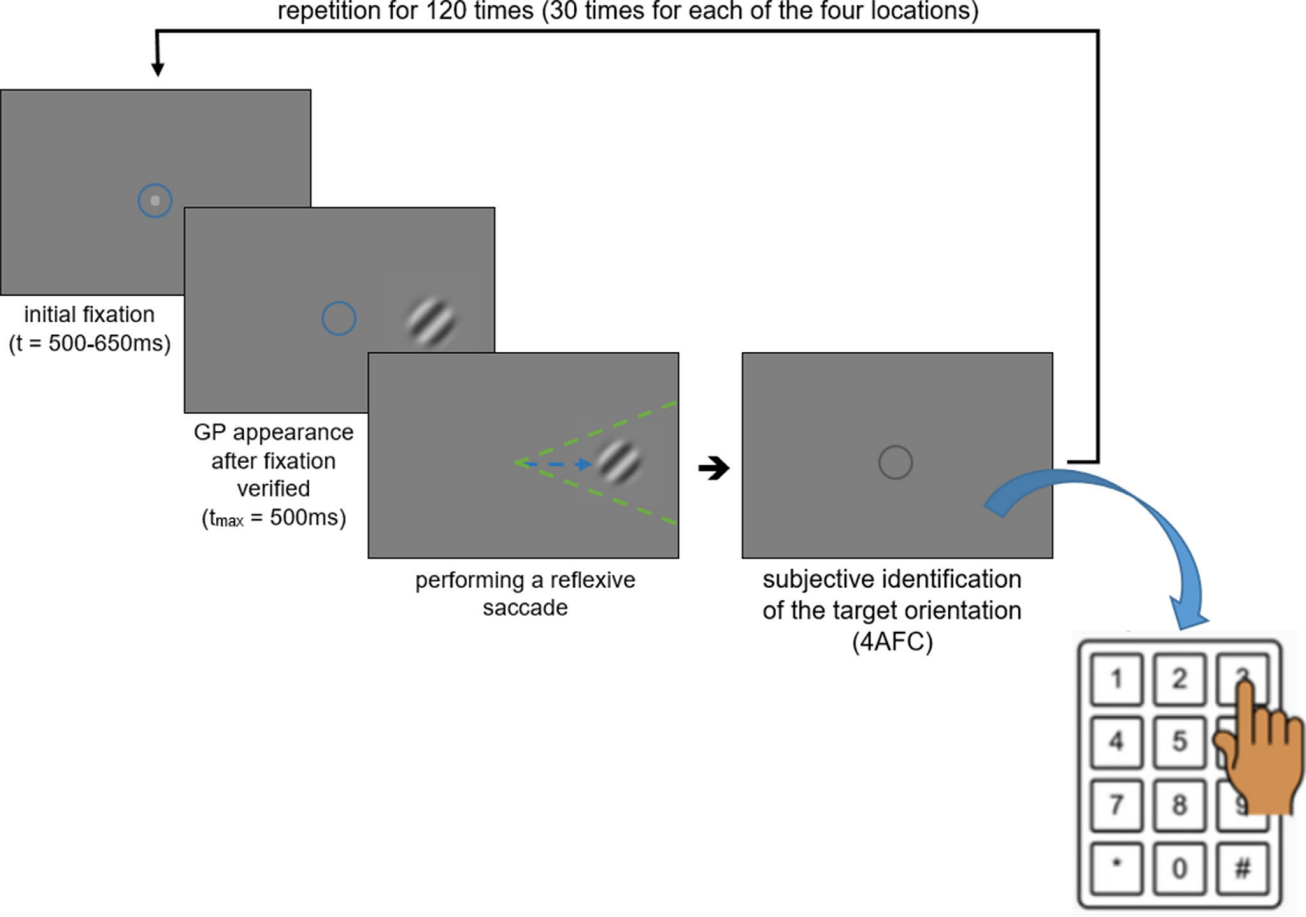
Procedure for the saccade-based measurements. The *blue circles* represent the area of fixation. The *blue arrow* illustrates the expected direction of a reflexive saccade. The *green dashed lines* show the cone within which the saccade was considered correctly oriented. When the *gray* *circle* was shown, the test required answering the grating orientation (in this example, the correct response was pressing 3 or 7 on the number pad).

The participants’ task was slightly different between the two types of measurement. All subjects performed both measurements in a randomized order. In the saccade-based measurement, participants were instructed to move their gaze toward the location where the target was identified (to try to catch the target with their gaze). After the eye movement or target expiration, subjects had to answer the orientation of the Gabor patch in a 4AFC (four-alternatives forced choice) paradigm, using a keyboard as depicted on Figure 1. For controlling the location-specific QUEST+ algorithm, correctly oriented saccades were taken as correct responses, or otherwise, if no or wrongly oriented saccade was detected. The keyboard-based responses to the grating orientation within this method were collected as additional data and did not influence the adaptive psychometric procedure. This data was only collected to better compare the CSFs of the saccade- and keyboard-based measurements, because there was a discrepancy in the fixational mark presence (in the keyboard-based measurement events, the fixation mark was present in the stimulus presentation phase to avoid reflexive saccades).

The second measurement method covered a purely subjective approach in which only the orientation of the target was asked in a 4AFC paradigm. Fixation had to be maintained on the central fixation mark, which was present also during the stimulus presentation phase. Equally to the first method, the initial fixation phase varied in time. Participants had to respond to the four available visual stimulus orientations with the keys 1 or 9, 2 or 8, 3 or 7, and 4 or 6, respectively. The correct answer to the spatial orientation of the stimulus was considered as “seen” or “not seen” otherwise. This response was used as input for QUEST+.

Each eccentricity and spatial frequency combination was tested with both measurement methods with 120 target presentations each. There were always 30 target presentations per direction, and every orientation of the grating was presented 30 times. The total number of trials in the saccade-based measurements was 132, from which 12 were catch-trials at which no target was present. However, they followed the same workflow to hide them among other trials.

### Application of the Adaptive Psychometric Procedures

The QUEST+ adaptive psychometric procedure^30^ was used to control the contrast level of the stimuli at each of the four VF directions (nasal, temporal, inferior, and superior) independently (four instances of the psychometric procedure were used) because CS varies between different visual field locations.^21^ As the psychometric function of the entropy-based adaptive psychometric algorithm, the cumulative Weibull distribution function was used with the parameters threshold, slope, upper and lower asymptote. The parameter space for threshold had 15 contrast levels ranging from ≈ 0.024% to ≈ 66.0%. The slope had a predefined parameter space of 0.5 to 5.5 in steps of 0.5 since it could differ among subjects and tested locations. Since the convergence of the entropy function could vary among subjects and tested locations, the number of grating presentations was fixed, resulting in the same amount of data for every participant.

### Analysis of the Acquired Data

To find the contrast threshold for the calculation of CS, the occurrence rate of reflexive saccades depending on the stimulus contrast level was fitted with a psychometric function using Psignifit^31^ in MATLAB. The cumulative Weibull distribution function was used as the fitting function with the following parametrization

(2) $$\begin{array}{c} \;\;\;\;\;\;\;\begin{array}{c} \Psi \left(x;m,w,\gamma ,\lambda \right)\;=\;\gamma \;+\;\left(1\;-\;\lambda \;-\;\gamma \right)\left(1-e^{\log\left(0.5\right)e^{c}\frac{\log(x)-m}{W}}\right) \end{array} \end{array}$$

with *c* = log(−log(0*.*05)) – log (−log(0*.*95)). The fitting parameters were guess rate *γ* and lapse rate *λ*, representing the lower and upper asymptote, as well as the threshold *m* and the width *w* between the 0*.*05 and 0*.*95 points. Both *m* and *w* are in log space of the stimulus parameter *x*. The relation between the width *w* and the slope *s* of the cumulative Weibull distribution function, as also used by QUEST+, is *s =*$\;\frac{c}{w}$ *.* The contrast threshold *CT* was calculated from *m* as *C* = *em*. Furthermore, the threshold was taken for every measured spatial frequency of the grating and was later converted to the CS value as *CS =*$\;\frac{1}{CT\;}\;$. For plotting of the data and statistics, the log_10_(*CS*) was used. At last, the CSF was created as a fit of the CS values depending on spatial frequency *SF* with a log-parabola, considering the ascending and descending part of the CSF as already suggested by Lesmes et al.,^32^ using the following fitting function:

(3) $$\begin{array}{c} \begin{array}{c} \;\;\;\;\;\;\;\;\;CS\left(SF\right)=\log_{10}\left(\gamma_{\max}\right)-\log_{10}\left(2\right){\left(\frac{\log_{10}\left(SF\right)-\log_{10}\left(SF_{\max}\right)}{\beta }\right)}^{2}, \end{array} \end{array}$$

with peak sensitivity *γ_max_*, peak spatial frequency *SF_max_*, and the function's bandwidth β.^32^ The CSFs were fitted individually for each subject and all tested retinal locations in the three eccentricity levels. Additionally, the mean CSFs were fitted, calculated across all participants for all tested locations, eccentricities and testing methods.

## Results

### Psychometric Fits

**Figure 2.**
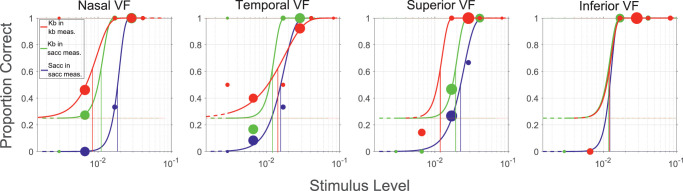
Four subplots are shown for the four tested visual field directions. Each subplot contains three psychometric functions for the three testing procedures. The *red* fits represent the keyboard-based responses in non-saccadic measurements, the *green* fits represent the keyboard-based responses in saccadic measurements, and the *blue* fits represent saccade-based responses in saccadic measurements. Please note that for the keyboard-based measurements, the lower asymptote (guess rate) is shifted to 25% because of the 4AFC paradigm. The dot size (area) scales with the testing frequency of the particular contrast level, as selected by the adaptive psychometric procedure. The shown data are one example measurement of one subject at spatial frequency 2*.*2 cpd) and eccentricity 2°.

The psychometric function fits for all three testing procedures (saccade-based responding in saccadic measurements, keyboard-based responding in saccadic measurements and keyboard-based responding in non-saccadic measurements) are shown in ^1^Figure 2 for one example subject.

### Correlation of the CS Values

**Figure 3.**
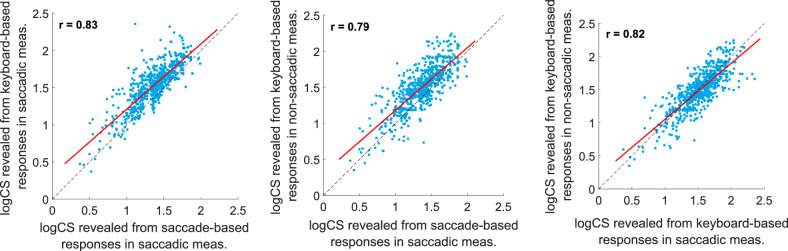
The three possible combinations of correlations are shown for the three testing methods. The *blue points* represent the logCS values for all tested subjects, directions, eccentricities and spatial frequencies. Please note the correlation coefficients are provided in each subfigure.

**Figure 4.**
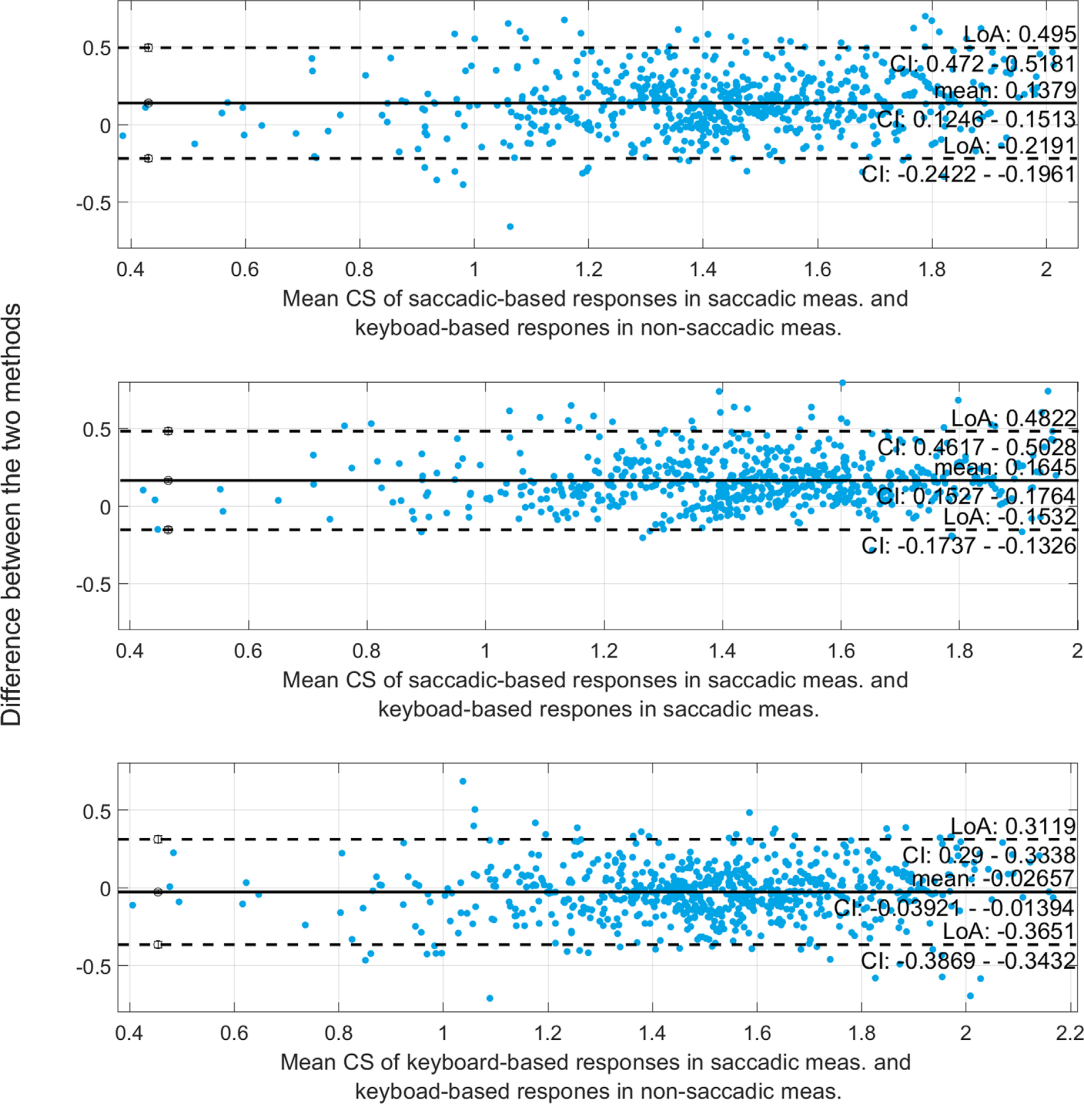
The blue points represent the logCS values for all tested subjects, directions, eccentricities and spatial frequencies. Please note that the confidence intervals (CI), mean value, and the lower and upper bounds are provided in the figure. These plots along with the correlation plots above were considered to yield a first insight about the possibility to use reflexive saccades as another type of eye movement for CS testing.

We created three correlation (Fig. 3) and Bland-Altman (Fig. 4) plots of the obtained CS values to compare the three measurement methods to have a first insight into the comparability of our data. Correlation of CS values is strong for all combinations of the three measurement procedures with correlation coefficients of *r* = 0*.*79 or higher. However, the Bland-Altman plots show an offset between the saccade-based responses in saccadic measurements and keyboard-based responses in non-saccadic measurements (0.1379) as well as between the saccade-based responses and keyboard-based responses in saccadic measurements (0.1645). The smallest offset was found between the two keyboard-based responses in saccadic and non-saccadic measurements (−0.0266). Friedman's test shows a significant effect of the testing procedure (χ^2^ (2159) = 482; *P* < 0*.*0001) with significant group-wise differences between all three approaches (*P*_all_ < 0*.*0001). The median CS for the three testing procedures was 1.39, 1.54, and 1.52 in the saccade-based measurements, in the keyboard-based responses in saccadic trials, and the non-saccadic keyboard-based measurements, respectively.

### Mean CSFs

**Figure 5.**
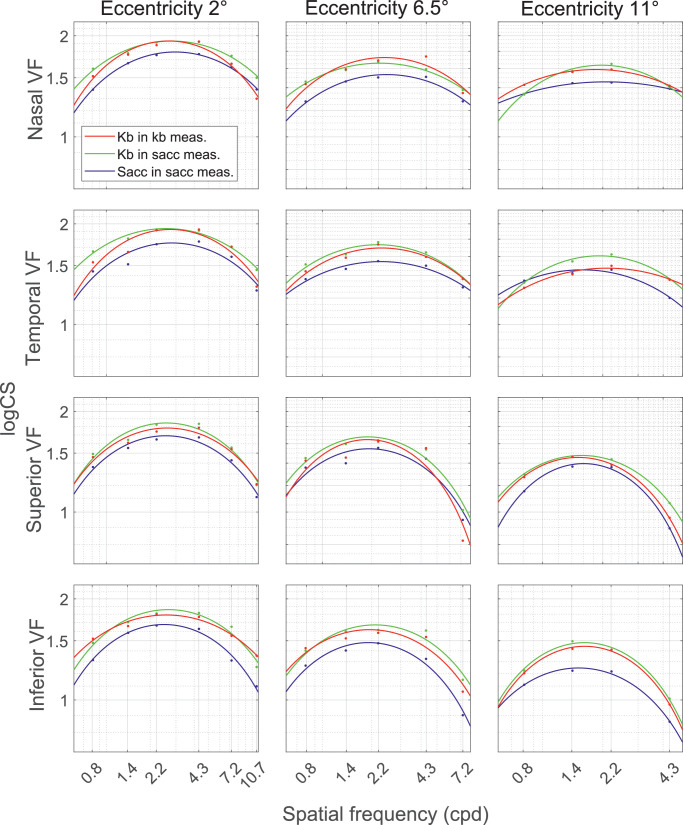
The mean CSFs of all subjects were fitted for every eccentricity level (column) and every visual field direction (row). The first column corresponds to the first eccentricity level (2°), second column to the second eccentricity level (6*.*5°) and the third column to the third eccentricity level (11°). Each subplot contains a *blue* CSF representing the saccade-based responses in saccadic measurements, *green* CSF representing the keyboard-based responses in saccadic measurements, and a *red* CSF representing the keyboard-based responses in non-saccadic measurements. Note that the ranges of spatial frequencies vary for every eccentricity level.

Mean CSFs (Fig. 5) were calculated over all participants for the three testing procedures and the three tested eccentricity levels, considering them as another way of comparing the three testing approaches besides the correlation plots described in the previous section. The results in the saccade-based measurements show slightly worse values of CS than the two other approaches, supporting the median outcomes presented in the previous section. Nonetheless, the trend of all the CSFs remained comparable across all testing procedures. Second, CS was better in the nasal and temporal visual field compared to the superior and inferior direction, with more profound differences for higher eccentricity levels and higher spatial frequencies. Furthermore, we statistically tested the effects of the method, eccentricity and direction on the fitting parameters of the mean CSFs (peak sensitivity, peak spatial frequency and the function's bandwidth). The three-way analysis of variance showed significant effect of the direction of the visual stimulus (F(3,32) = 16.97; *P* < 0*.*0001) eccentricity (F(3,33) = 256.78; *P* < 0*.*0001) and method (F(3,33) = 60.01; *P* < 0*.*0001) on the peak sensitivity. The peak spatial frequency is significantly influenced by the direction of the visual stimulus (F(3,32) = 20.45; *P* < 0*.*0001) and eccentricity (F(3,33) = 187.00; *P* < 0*.*0001) but not by the method (F(3,32) = 0.01; *P* = 0.99). Finally, our data show a significant effect of the direction of the visual stimulus (F(3,32) = 7.26; *P* = 0.0009) and eccentricity (F(3,33) = 3.69; *P* = 0.038), yet the method continued to have an insignificant effect (F(3,33) = 1.63; *P* = 0.21) on the function's bandwidth.

### Individual Visual Field Location-Specific CSFs Measured With Reflexive Saccades

**Figure 6.**
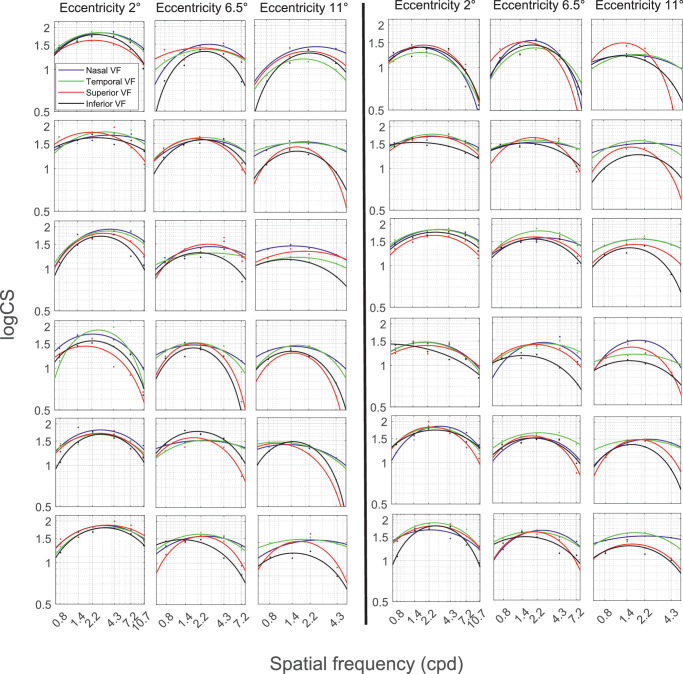
The location-specific CSF for the saccade-based measurement is presented for each participant (row) and eccentricity level (column). Similar to the previous figure, each subplot shows a *blue* CSF representing the nasal VF, *green* CSF for the temporal VF, *red* CSF representing the superior VF, and a *black* CSF for the inferior VF.

Figure 6 shows the CSFs of saccade-based measurements for all participants for the four directions and the three eccentricity levels. We also present median and mean CS values with standard deviation in the Table, calculated for every combination of direction and eccentricity. Furthermore, we tested the effect of the spatial frequency and the direction on CS for the three eccentricity levels individually. The two-way analysis of variance revealed a significant effect of the spatial frequency and the direction on CS for the first (F(5,282) = 41.58; *P* < 0*.*0001, F(3,284) = 4.93; *P* = 0.0024), second (F(4,235) = 55.69; *P* < 0*.*0001, F(3,236) = 10.18; *P* < 0*.*0001) and third (F(3,188) = 40.39; *P* < 0*.*0001, F(3,188) = 30.11; *P* < 0*.*0001) eccentricity level. The interaction of the two variables was not significant on the first eccentricity level (F(15,272) = 0.49; *P* = 0.94); however, the significance was shown for the second (F(12,227) = 2.56; *P* = 0.0034) and third (F(9,182) = 3.03; *P* = 0.0022) eccentricity. On top of that, the 2160 catch-trials had a false-positive rate of 4.3%, with 93 catch-trials in which the gaze exceeded the fixation area although no target was present.

**Table.** Median (Mean ± SD) of CS Values Were Calculated Over All Subjects and Spatial Frequencies, Individually for the Three Eccentricity Levels And The Four Visual Field Directions

	Eccentricity 2°	Eccentricity 6*.*5°	Eccentricity 11°
Nasal VF	1.57 (1.52 ± 0.27)	1.41 (1.38 ± 0.18)	1.38 (1.36 ± 0.14)
Temporal VF	1.51 (1.51 ± 0.29)	1.42 (1.40 ± 0.19)	1.36 (1.31 ± 0.18)
Superior VF	1.47 (1.44 ± 0.29)	1.38 (1.32 ± 0.26)	1.21 (1.19 ± 0.23)
Inferior VF	1.44 (1.40 ± 0.27)	1.26 (1.25 ± 0.27)	1.11 (1.11 ± 0.19)

## Discussion

The current study aimed to establish and validate a novel approach of objective assessment of peripheral CS based on eye movements, extending already conducted research on eye movement-based central CS testing.^1^^,^^3^^,^^4^^,^^6^ For our testing, we used reflexive saccades, a visually-guided eye movement occurring toward a novel stimulus in the visual field of an observer.^13^ Our test also extends the portfolio of saccade-based tests, containing for instance the SVOP technique,^14^^,^^15^ for peripheral visual performance examinations. Such a set of tests may help to examine noncommunicative participants^1^ or children.^15^ In our study we presented a visual stimulus (Gabor patch) defined by a spatial frequency and eccentricity level in four possible directions in a random order. In our novel test, the task for the participant was to perform a saccade toward the stimulus (to catch the target with their gaze). To validate our newly proposed peripheral CS test, saccade-based measurements were compared to an established subjective test based on a response to the orientation of the stimulus. In this non-saccadic subjective measurement, a stable centrally-located fixation mark was present, leading to possible differences in attention between the two measurement events. To study this possible influence, the subjective judgement was also performed directly in the saccade-based measurement by identifying the orientation after each trial. A significant difference was found between the two keyboard-based procedures (CS measured by the keyboard-based responding in the saccadic measurements performed better). However, this difference is small (mean difference 0*.*027), and therefore we consider it irrelevant for clinical testing. On top of that, the Bland-Altman plots showed the highest agreement between the two keyboard-based procedures. In contrast to a previous study by Rosen et al.,^21^ we randomized the target directions and orientations within one eccentricity level and spatial frequency in every measurement to minimize the potential influence of attention.^33^ Moreover, we implemented catch-trials, containing no visual stimulus. In 4.3% of catch-trials a saccade was detected. A previous study on accuracy and precision of saccades showed at least 9% of catch-trials containing a saccade.^34^ Vingrys et al.^35^ stated that a false-response rate should be lower than 20% to consider a visual field test as reliable.^35^ Therefore we consider our false-response rate low and our test to be solid. Nonetheless, we cannot report on the reason for our false-response rate because our saccadic detection did not consider the landing point (the amplitude). A potential explanation could be the occurrence of large fixational eye movements exceeding the central fixation area (1°). As found by Krejtz et al.^36^ or Martinez-Conde et al.,^37^ events of fixational eye movements larger than 1° are rare; hence, only a small amount of our catch-trials was found as false-positive. In future clinical testing, catch-trials might be used as a verification of the test reliability in individual subjects, as already suggested before.^35^

The results of the current study show first a strong correlation of CS values obtained from the saccade-based measurements and both types of keyboard-based responses, whereas all correlations calculated indicate that the different response types are highly comparable. However, the saccade-based measurements generally show slightly worse performance in CS testing than both keyboard-based measurements. This offset is shown by the Bland-Altman plots comparing the CS of saccade-based responding to both keyboard-based procedures. One reason for this finding could be the difference in the psychometric testing method (4AFC vs. yes-no-task), leading to different guess rates in the fitting procedure (guess rate set to 25% in both keyboard-based responses, while set to zero in the saccade-based responses).^38^ Furthermore, it might be the case that sometimes, in low-contrast testing (high CS), the contrast of the visual stimulus was too low to trigger a reflexive saccade, even if the target was judged correctly for its orientation. Another possible reason for the saccade-based responses to underperform the other two measurement procedures could be that the latency of the saccadic onset was longer than the stimulus presentation time, because the onset time was found to be contrast-dependent.^18^

Moreover, the current study found a systematic decrease of CS with increasing eccentricity, as was hypothesized already after the outcomes of a previous study testing other eccentricity levels.^21^ Our study confirmed better CS in the horizontal visual field over the vertical one in both eye movement-based and keyboard-based trials. However, our test's eccentricity levels were smaller than in other studies.^21^^,^^39^^,^^40^ Here we also consider the maximum eccentricity we reached in our study (11°) as a limitation of our set-up, because the further eccentricities would have provided relevant information of patient's peripheral visual performance, particularly in early-detection procedures of various eye diseases. For testing larger eccentricity levels, our test would require a larger screen or performing the examination using a projector for instance. From previous research, better CS is expected in the inferior VF compared to the superior VF. Our study could confirm this only in keyboard-based measurements, whereas saccadic trials show an opposite trend. This finding could be explained by nonuniformity in triggering vertical saccades. For example, Abegg et al.^41^ found shorter latencies for upward oriented saccades than downward oriented ones. On top of that, Tzelepi et al.^42^ suggested that stimuli in the upper and lower visual field may have different impacts on areas of the visual system related to visual attention and motor preparation, concluding behavioral asymmetries in ocular motor performance.

Nonetheless, the previous research showed a successful assessment of the patient's VF using reflexive saccades.^15^ We suggest that such testing procedures are also suitable for remote screening using, for instance, a VR headset, as already done by Tatiyosyan et al.^5^ for optokinetic nystagmus-based CS examinations. Although there are some considerable technical limitations, as for instance the eye-tracking latency^43^ or its quality,^44^ the VR headsets provide a bigger field of view for possible testing of larger eccentricities,^45^ compared to our setup. Testing further eccentricities would be useful in the early detection of several eye diseases such as glaucoma. Our test design should support such testing because the direction of a saccade has been calculated right after exceeding the fixation area (no need for high accuracy of the eye tracker in the periphery).

## Conclusion

The current study shows a possible approach of objective and fully automated estimation of CS in selected areas of the visual field. We found a strong correlation of the CS values between the newly developed method and an established procedure. On top of that, the current study showed a successful replication of the CSF across various visual field locations. Hence, this study indicates the possibility of using reflexive (visually-guided) saccades to assess visual performance over the whole visual field objectively, not only considering CS but also visual acuity or the actual size of the visual field.
